# Autopoiesis, Thermodynamics, and the Natural Drift of Living Beings: Another Way to the New Evolutionary Synthesis

**DOI:** 10.3390/e24070914

**Published:** 2022-06-30

**Authors:** Mario Villalobos, Ramiro Frick, Sergio Vicencio-Jimenez

**Affiliations:** 1 Escuela de Psicología y Filosofía, Universidad de Tarapacá, Arica 1010069, Chile; 2 Department of Philosophy, Universidad Alberto Hurtado, Santiago 6500620, Chile; ramirofrick@gmail.com; 3 The Center for Hearing and Balance, Department of Otolaryngology-Head and Neck Surgery, Johns Hopkins University School of Medicine, Baltimore, MD 21231, USA; svicenc1@jhmi.edu

**Keywords:** modern evolutionary synthesis, autopoietic theory, natural drift theory, new evolutionary synthesis, teleology, thermodynamics

## Abstract

The New Evolutionary Synthesis (NES) groups a series of theories that, departing from the gene-centric approach of Modern Synthesis evolutionary theory (MS), place the organism as the central agent of evolution. Two versions of NES, each one with advantages and disadvantages, can be distinguished in this regard; the restrictive NES and the comprehensive NES. Comparatively, the comprehensive NES is a more robust theoretical construction than the restrictive one because it comes grounded on a general, thermodynamically informed theory of living beings (something that the restrictive NES lacks). However, due to its strong teleological commitments, the comprehensive NES has serious problems fitting with modern science’s methodological framework; a problem that the restrictive version, with no explicit commitment to teleology, does not face. In this paper, we propose the autopoietic approach to evolution as a way of integrating these two versions of NES, combining the theoretical robustness of the comprehensive view with the methodological appropriateness of the restrictive one. The autopoietic approach, we show, offers a non-teleological, organism-centered theory of evolution, namely the natural drift theory (NDT), and a grounding on a thermodynamic theory of living beings, namely the embodied autopoietic theory (EAT). We conclude that, from the programmatic point of view, an autopoietic (NDT plus EAT) approach to evolution offers a promising way to develop the NES project.

## 1. Introduction

The dominant paradigm in evolutionary biology, sometimes labeled ‘standard’, ‘traditional’, ‘Neo-Darwinian’, or most frequently, ‘Modern Synthesis’ (MS) evolutionary theory, is under severe questioning [1,2,3]. The main points in the discussion have to do with MS’s reductionist character, specifically with its gene-centric approach to biological phenomena such as development, heredity, reproduction, and evolution. Briefly stated, in MS’s view the organism’s development is reduced to the ‘reading’ or expression of a genetic program. Reproduction and inheritance are reduced to mechanisms of DNA copy and transmission. The origin of variation is reduced to the occurrence of genetic mutations, and the evolutionary process itself is reduced to a change in allele frequencies in a population over time. As Walsh critically points out, “Modern Synthesis evolution is a fundamentally molecular phenomenon” ([4], p. x).

In response, critics have called for a rethink of the evolutionary theory, arguing for the elaboration of a new evolutionary synthesis (e.g., [5,6,7]), more holistic and focused on the organism and its constructive interaction with the environment. Several theoretical streams converge in this project, including among others the integrated evolutionary synthesis [8,9], the extended evolutionary synthesis [10,11] the situated Darwinism [4,12], and the fourth perspective on evolution [13]. These critical streams come with different emphases and degrees of radicality but share the spirit of building an alternative research program to MS, putting the organisms, not the genes, as the central agents of evolution [14]. For the purposes of this paper, the generic expression ‘new evolutionary synthesis’ (NES) will be used to collectively designate all these new trends in contemporary evolutionary biology.

The NES movement implies reconceptualizing the organisms as agents. However, the precise sense and reach of this reconceptualization are not uniform within the movement [13]. Two different versions of NES can be distinguished in this regard. One version, which we call restrictive, sees the organisms as agents in a weak, restricted, and non-teleological sense, without appealing to, or being grounded on, any general theory of living beings in an explicit way. The other, more ambitious one, which we call comprehensive, sees the organisms as agents in a strong, comprehensive, and teleological sense -what they call the agent theory-, appealing as a ground to a thermodynamically inspired theory of living beings that conceives of them as natural purposes.

In this paper, we argue that in between these two versions of NES there is room for a third and better one, which we identify with the autopoietic approach to life and evolution. This approach contemplates the natural drift theory of evolution (NDT) and the embodied autopoietic theory of living beings (EAT). The rationale behind our argument is as follows. Grounding NES on a thermodynamic theory of living beings, as the comprehensive version does, is positive because it ontologically strengthens the project in front of MS. However, linking such a grounding to teleology, as the comprehensive version also does, is highly problematic because it puts NES in conflict with modern science’s methodological framework. Comparatively, the restrictive NES, lacking an ontological grounding, is a theoretically weaker version than the comprehensive one, but, having no commitment to teleology, fits well with the framework of modern science. In this scenario, we propose the autopoietic combination NDT+EAT as an intermediate way that can integrate the positive points of the two existing versions of NES, avoiding their weaknesses. NDT, as we will show, is an organism-centered evolutionary theory that provides NES with a teleology-free, scientifically respectful ontological grounding in the form of a thermodynamic, embodied autopoietic theory of living beings (EAT).

It is important to clarify from the start the nature and reach of our argument. Our analysis is theoretical, philosophical, and programmatic. We will not argue that the autopoietic approach, i.e., NDT+EAT, is a better option because it delivers new predictions or greater empirical content relative to the existing versions of NES. We assume, for the sake of the argument, that the autopoietic approach, given its core theoretical assumptions (see Section 3), has at least a similar empirical power to the existing versions of NES. On that common ground, we argue that the combination NDT+EAT, from the programmatic point of view, is a better option because, although it provides NES with an ontological ground, it does it in a more parsimonious, less philosophically demanding way, avoiding methodological conflicts with modern science.

The structure of the paper is as follows. In Section 2, we characterize the NES project and distinguish its restrictive and comprehensive versions, highlighting their respective advantages and disadvantages. In Section 3, we introduce the autopoietic framework and its NDT as an evolutionary approach that meets the core ideas of NES. In Section 4, we examine how an embodied autopoietic theory of living beings (EAT) can provide a thermodynamic and yet non-teleological ground for NDT, and by extension, for NES. Finally, in Section 5, we recapitulate the main points of the argument and briefly discuss some implications.

## 2. NES and Its Two Versions: Advantages and Disadvantages

NES can be characterized, basically, by contrast with MS. The different way, relative to MS, in which NES understands key concepts such as inheritance, development, evolution, and organism, defines NES’s theoretical identity. (In the next section, we will argue that NDT is a NES representative by appealing to these key concepts). Let us briefly review the core assumptions of NES.

### 2.1. NES’s Core Ideas

*Inheritance.* A central idea in NES is the emphasis on extended or inclusive inheritance [15]. In contrast to MS’s notion of heredity, which is restricted only to genetic inheritance, in NES heredity is defined to include all causal mechanisms by which offspring come to resemble their parents [10]. Inheritance extends beyond genes to encompass epigenetic inheritance, physiological inheritance, ecological inheritance, and behavioral-cultural inheritance [16,17,18].

*Development.* In MS, development is understood as the execution of a program encoded in and inherited through the genes, where the rest of the developing organism operates as a decoding machine. In contrast, in NES phenotypes themselves are not inherited but rather re-constructed in development, and development is understood as a process that depends not only on genetic factors, but also on epigenetic, environmental, behavioral, and cultural [10,19,20,21].

*Evolution.* Unlike MS, in which evolution only requires, and is often reductively defined as, change in gene frequencies in populations [22], in NES there is a “broadened notion of evolutionary process” that includes all forms of inheritance ([10], p. 2). On the recognition that there is more to heredity than DNA, for NES “evolution can no longer be defined in terms of changes in gene frequencies” ([23], p. 38). Thus, in NES evolution is understood more broadly as a “transgenerational change in the distribution of heritable traits of a population” ([10], p. 2).

*Organism.* In contrast to MS′s gene-centrism, NES advocates for an organism-centered perspective. In MS, organisms are usually understood as passive entities whose characteristics are determined by the genes they carry and by the selective pressures imposed by the environment to which they adapt. In contrast, in NES organisms are active entities, and their activities are an important part of the causes and explanation of their own development, of the characteristics of the environment in which they inhabit, and of their own evolution [24]. This involves, but is not restricted to, the idea of ‘niche construction’ which is taken to be an important biological process neglected for a long time in evolutionary biology [25]. In the words of Nicholson:

“[T]he emerging view of evolution dislodges the gene from its privileged position and restores the organism back to the center of the evolutionary stage. In this organism-centered view, organisms rather than genes are the primary *agents* of evolutionary change”.

([24], p. 349. Emphasis added)

Crucially, these core ideas, and specially the last one, imply conceiving of the organisms as the agents of evolution. And here is where we find a key difference between the restrictive and comprehensive versions of NES.

### 2.2. The Restrictive and the Comprehensive Versions of NES

In the restrictive version of NES, the agential view aims to recognize the active and central role of the organisms in relation to their environment and evolution. Evoking a line of thought that can be traced at least to Lewontin [26], it is argued that organisms are not passive entities that adapt to a given environment but rather constructive agents that shape the very environment they inhabit [25,27,28]. Complementarily, it is argued that organismal-level phenomena are central, not peripheral explanatory factors in evolutionary change, and that therefore evolution cannot be reductively explained only (or mainly) in terms of genes. Herrington and Jablonka, for instance, express this view when they talk about “the importance of the organism (…) as an ‘agent’ of evolutionary change [in the sense that] genes are followers of life’s evolutionary processes rather than its drivers or instructors.” ([29], p. 374. Original brackets removed).

Note that this agential view is restricted to and concerns only evolutionary theory. It does not appeal to any general theory of life. That is, it does not come explicitly committed to, or backed up by, any ontological theory or definition about what living beings are. It asks us to recognize organisms as constructive and central factors in the evolutionary process, but not to recognize them, additionally, as purposive entities. In doing so, this view conflicts with some aspects of MS (passive adaptationism and gene-centrism) but with nothing beyond that. It poses, so to speak, an internal challenge to evolutionary theory.

All this contrasts with the comprehensive version of NES, where we find a philosophically loaded sense of agency that, as we will see, conflicts not with this or that aspect of MS but with biology and natural science as such. In this view, organisms are taken to be, literally, agents; that is, entities that have, and act according to, ends and purposes [13]. This view comes as an ontological claim that characterizes living beings in teleological terms, grounded on a general theory of living beings that goes beyond the evolutionary terrain, namely the autonomy theory of life [30,31,32,33]. (We follow Barandiaran [34] in grouping under the same generic denomination theories of autonomy that come from different fields and disciplines, such as the organizational approach in theoretical biology and the enactive one in cognitive science). Following the teachings of this theory, advocates of the comprehensive view argue that “organisms are fundamentally purposive entities” ([4], p. ix), and that said characteristic needs to be recognized if we really want to account for the active role of organisms in evolution [13,35]. (The reasons that motivate this teleological requirement will be reviewed in the next section. See specifically Section 3.2).

The conflict of this teleological view with biology and natural science is deep and well-known. As the proponents of this view themselves recognize, biologists “have an animadversion to purpose” ([4], p. ix), and, as a general norm, “natural purposes and teleology are (…) repugnant to scientists and philosophers of a naturalistic bent” ([4], p. 188).

In front of this animadversion, far from backing down, the representatives of the comprehensive view answer by building an “agent theory” ([4], p. 212) that attempts to “rehabilitate the notion of natural purpose” within science ([4], p. 164). To this end, they recur, as we said, to the autonomy theory of living beings [32], which offers a naturalization strategy of teleology by appealing to the thermodynamic organizational properties of life (more on this in Section 4). Validating teleology within natural science is indeed a crucial step in this version of NES since the whole project of an organism-centered evolutionary theory is taken to depend on that. In the emphatic words of Walsh, the “nascent organism-centered evolutionary thinking will be nipped in the bud if it doesn’t find a way to reconcile modern scientific methodology with teleology” ([4], p. 188).

With these two versions in mind, the question naturally arises as to which path NES should follow. If NES aims to develop and consolidate as an alternative to MS, which version should it pursue? Compared to the restrictive version of NES, the comprehensive view has the advantage of being supported by a general theory of living beings, namely the autonomy theory. It is, in that sense, a theoretically more substantive and robust proposal. Its disadvantage, however, is that its teleological commitment imposes a heavy and risky conditional on the future of NES; either the project finds a way to naturalize teleology, reconciling it with science (a far from easy feat), or it dies. The restrictive version of NES, on the other hand, is comparatively weaker or shallower than the comprehensive one; it lacks a theoretical ground in terms of an ontological conception of living beings. However, it has the advantage that, since it does not commit to a teleological view, fits frictionlessly into the methodological framework of science, avoiding the strong conditional imposed by the comprehensive view.

It would be optimal if we could find a way to combine the advantages of the restrictive and comprehensive versions of NES without suffering their disadvantages. Namely, if we could find a comprehensive alternative to MS that fits seamlessly into the modern scientific framework. Is that possible? Walsh, one of the leading representatives of comprehensive NES, sees a major obstacle to this task and offers a solution at the same time:

“The principal impediment to generating a comprehensive alternative to the Modern Synthesis has been the modern antipathy to natural purpose. (…) [O]ur post-Scientific Revolution deteleologised worldview has conspired against the inclusion of organisms in evolution. [O]rganisms participate in evolution as agents. In order to accommodate this fact we need a special kind of theory, that I call an ‘agent theory’”.

([4], p. xii)

A few critical comments about this diagnosis. It is true that modern science expresses an antipathy to natural purposes, but has that been the principal impediment to generating a comprehensive alternative to MS? Perhaps the problem is different, namely the lack of an NES that, being comprehensive, respects modern science’s non-teleological framework.

Modern science establishes indeed a deteleologised worldview, as Walsh correctly observes, but that does not seem to conspire per se against the inclusion of organisms in evolution. The anti-teleological stance of modern science becomes a problem only if such an inclusion presupposes a commitment to teleology. But, is NES forced to make such a commitment?

We agree with Walsh that organisms participate in evolution as agents, but do not think that to accommodate this fact we need a “special kind of theory,” an “agent theory” in the teleological sense that Walsh and the comprehensive view promote. In our view, what we need is a comprehensive theory that, both recognizes organisms as agents of evolution and respects modern science’s framework. Can we have such a theory?

In the next section, we will introduce a theory that we think meets these requirements, namely the autopoietic theory of living beings and evolution. This theory, as we will show, embraces the core ideas that NES promotes, grounds such ideas on a comprehensive theory of living beings, and holds a non-teleological view of organismic agency.

## 3. The Autopoietic Approach to Evolution: A Third and Better Way to NES

In this section, we will show that the autopoietic theory of evolution, known as natural drift theory (NDT) [36,37], has all the characteristics needed to open a third and better way to NES. These characteristics are:

(i) to be an ontologically grounded theory of evolution,
(ii) to respect the non-teleological framework of natural science, and
(iii) to embrace the core assumptions of NES.

For those who are somewhat familiar with the autopoietic tradition, points (i) and (ii) might be relatively easy to appreciate (they will be briefly commented). Point (iii), however, will merit a more extended analysis. Demonstrating that NDT is compatible and aligned with the NES depends, to some extent, on the version of the NES we are dealing with. Against the restrictive NES, being a more focused and less ambitious version than the comprehensive one, it suffices to show that there are relevant coincidences between NDT and the NES on key concepts of evolutionary theory. In the face of the comprehensive NES, however, this is not enough. The comprehensive NES defends its teleological conception of agency by appealing to certain theoretical insights that presumably (only) such a conception would deliver or facilitate. Here, NDT needs to demonstrate that, despite its non-teleological basis, it is capable of delivering or facilitating the same theoretical insights that the comprehensive NES considers relevant.

Let us start by briefly commenting on points (i) and (ii). NDT is an ontologically grounded theory of evolution in which the foundation is provided directly by the autopoietic theory of living beings. Actually, NDT arises out of “the consequences that (…) the notion of autopoiesis has for the understanding of the phenomena of reproduction, evolution and heredity” ([38], p. 46).

We will unpack such consequences soon when addressing point (iii) in Section 3.1, but first, it is important to appreciate the ontological dimension of the theory. The notion of autopoiesis characterizes living beings as self-producing natural systems [39,40,41]. In doing so, the theory recognizes organisms as being natural systems of a very particular kind, i.e., those that are capable of self-construction, but at the same time—and this is crucial for the topic of teleology—as being strictly and nothing more than that; natural systems. The autopoietic theory reasons that if natural systems in general (e.g., planets, stars, rivers, volcanoes, etc.), when studied scientifically, are not conceived teleologically, then, living beings, being also natural systems, should not be so conceived either [42,43]. According to the autopoietic theory, “living systems, as physical autopoietic systems, are purposeless systems” ([41], p. 86).

This non-teleological approach occupies an important place in autopoietic theory. For instance, in the seminal work *Autopoiesis and Cognition*, a separate section was explicitly dedicated to expose the anti-teleological arguments of the theory (cf. “II. Dispensability of teleonomy. 1. Purposelessness.” [41], p. 85–87). Since then, the rejection of teleology has been a constant in the theory [44,45,46] and has been reinforced in more recent works [43,47,48].

We will not unpack the anti-teleological arguments of the theory here, nor will we assess their soundness. Our point is not to demonstrate that the autopoietic theory, or natural science for that matter, is correct in conceiving of organisms in non-teleological terms, but rather to show that the autopoietic ground of NDT accords with and respects the non-teleological framework of natural science (that the defenders of the comprehensive version of NES demand to change).

From such a non-teleological ontology, the autopoietic theory extracts NDT; an evolutionary approach that, as it has been already observed by some commentators [49,50], aligns with NES’s general spirit.

### 3.1. Natural Drift Theory: A NES Theory

Several decades ago, the autopoietic theory elaborated NDT, which advanced some of the key ideas of the NES program [36,37,38,41]. As Raimondi observes, “[s]ince the early 1970s, the autopoietic approach has challenged genocentrism” ([50], p. 513) and argued for the centrality of the organism in evolution (see also [49]). The autopoietic theory sets the organism at the center of the biological theory (i) with respect to evolution itself, in the sense of giving primacy to the existence of the organism over the phenomenon of evolution (first organism, then evolution), and (ii) within evolutionary theory, in the sense of rejecting the reductionist gene-centric stance of MS, placing the organism and its behavior as genuine evolutionary factors. What concerns us here is this latter sense. (The first sense is collected in the autonomy theory [32], but does not feature, at least in an explicit way, in NES).

Broadly stated, NDT understands evolution as a historical process of structural variation with conservation of autopoiesis and adaptation through the systemic reproduction of (what the theory calls) the “*ecological organism-niche unity*” ([47], p. 640. Original emphasis; [51]). NDT provides several interesting and far-reaching implications for evolutionary biology, but for our purposes, here we will focus on those points that are relevant to the NES project.

The alignment between NDT and NES can be clearly appreciated if we draw a parallel with those key concepts that, as we saw in Section 2.1, define the NES project in opposition to MS.

*Inheritance.* Against MS, and in accordance with NES, NDT assumes that “heredity is a systemic phenomenon and not a molecular one” ([37], p. 293. See also [36,38]). In more general terms, what is conserved in reproduction, according to NDT, is the ecological organism-niche unity as a whole and not some particular component of it [47]. This systemic view, we take it, aligns with NES’s ideas of inclusive inheritance [10] and extended heredity [16].

*Development.* According to NDT, the process of organismal development or epigenesis is not predetermined by the initial structure of a living being (including its genome); rather, it arises “in the interactions (…) of the living system and the medium in which it lives” ([37], p. 269). This understanding of development, we submit, is at the same time contrary to MS’s preformationist metaphors of a genetic blueprint or programme [11], and akin to NES’s idea of “constructive development” ([10], p. 5).

*Evolution.* In NDT, evolution is viewed as a process of generation and differentiation of “relational configurations between organism and medium (…), not [of] genetic configurations per se” ([37], p. 290). This view clearly contrasts with MS’s gene-centric approach, and accords with NES’s broadened notion of evolution [10,29].

*Organism.* NDT places the organism’s “behavior [as] the central agent [of] evolution” ([37], p. 295). More explicitly, in NDT “it is the behavior of the organisms (…), and not their genetic constitution, nor any kind of directional external pressure (…) what guides (…) the course [of] evolution” ([37], p. 279). This perspective is at odds with MS’s passive and gene-centric view of the organisms, and again fits nicely with NES’s idea that “organisms (…) are the primary agents of evolutionary change” ([24], p. 349).

So far, and as long as the restrictive version of NES is concerned, we can say that NDT aligns with the NES program. However, as we observed before, the comprehensive version of NES sets a more demanding criterion than the restrictive one.

### 3.2. Natural Drift Theory: A Comprehensive NES Theory

Here we need to examine two additional argumentative lines advanced by the comprehensive view. The first one comes as a kind of bold, transcendental argument in which the teleological conception of agency, i.e., the agent theory [4,12], is claimed to be necessary to recognize the centrality of organisms in evolution [52]. Walsh explains:

“[M]odern synthesis evolutionary biology doesn’t recognize the contribution of organisms to evolution (…). [O]rganisms participate in evolution as agents. Their contribution to evolution *can only* be adequately captured by an evolutionary *agent theory*”.

([12], p. 169. Emphasis added)

In reply, we observe that while it is true that MS does not recognize the contribution of organisms to evolution, and that they, the organisms, are the ones that should be viewed as the primary agents of evolutionary change, it does not follow from that that the only way to capture this latter point is by adopting the agent theory promoted by the comprehensive NES. For we have just shown above that it is possible for a non-teleological approach to organisms, such as the autopoietic approach and its NDT, to adequately capture the contributions of organisms to the evolutionary process.

The second, subtler argumentative line does not hold that the teleological conception of organisms is necessary to obtain a proper understanding of evolution. It only claims that such a conception facilitates certain key insights that are relevant for evolutionary theory. This is the line we find, for instance, in [13]. According to Jaeger [13], the advantages of an agent theory can be appreciated if we ask ourselves what happens to evolutionary theory if we take organismic agency “at face value” ([13], p. 27). Following Walsh [4], Jaeger identifies three major implications:

“First, evolution must be treated as a fundamentally ecological or relational phenomenon (…). Second, it is not possible to causally separate the processes of inheritance, reproduction, and development (…). Third, there is no privileged control by replicator genes: genetic causation always has to be interpreted in its organismic context”.

([13], p. 27)

We might agree that agent theory indeed delivers these major implications for evolutionary theory. However, the relevant point for our argument is whether such implications can also be obtained in a philosophically cheaper way, by means of a non-teleological theory of organisms such as the autopoietic approach and its NDT. Let us briefly review the case.

First, regarding the idea of evolution as an ecological/relational phenomenon, in NDT it is the very unit of evolutionary analysis that is defined in terms of an “*ecological organism-niche unity*” ([47], p. 640. Original emphasis). As we saw in the previous section, for NDT evolution is the process of generation and differentiation of “relational configurations between organism and medium” ([37], p. 290).

Second, regarding the non-separability of evolutionary factors, NDT asserts that “heredity occurs (…) through systemic reproduction; and (…) that systemic reproduction occurs as a process (…) that involves organism and medium” ([37], p. 293). Thus NDT recognizes, as Jaeger demands, inheritance, reproduction, and organismal development as interconnected, non-separable processes.

Third, regarding the contextual causal role of genes, NDT submits that the realization of a living system involves the participation of all its components, and that “it cannot be claimed that any of them can alone be by itself responsible for its characteristics” ([37], p. 269). More specifically, for NDT “it is not possible to speak with property of genetic determinism, or to say (…) that certain features (…) are genetically determined” ([37], p. 269).

In summary, as well as explicitly keeping out purpose and teleology, NDT delivers equivalent theoretical consequences pointed out by [13] regarding the ecological/relational character of evolution, the non-separability of evolutionary factors, and the organismic contextual effects of genes.

NDT, we conclude, is a strongly organism-centered perspective compatible and aligned with the NES project. More than that, NDT offers a better way to develop NES in comparison to the restrictive and comprehensive versions currently available. The restrictive version lacks an ontological grounding regarding the nature of organisms. The comprehensive version has such a grounding, but given its teleological commitment, it conflicts with the scientific methodological framework. NDT, in delivering the theoretical insights that are relevant for NES, is an ontological, grounded, and yet non-teleological theory that, per the comparative analysis, offers a better way to NES.

## 4. Embodied Autopoietic Theory (EAT): A Non-Teleological Thermodynamic Grounding for NES

In the previous section, we argued that the autopoietic approach to evolution offers a non-teleological alternative for a comprehensive NES. On the one hand, we claimed that the autopoietic theory provides a teleology-free ontology of living beings. On the other, we showed that, in the form of NDT, the theory delivers, without teleology, the same theoretical insights as the comprehensive view regarding evolution. However, representatives of the comprehensive view might question the non-teleological credentials of the autopoietic approach, and by implication, of NDT, on the following basis.

The comprehensive NES appeals as a ground to the autonomy theory of living beings, which claims that teleology emerges as a natural property from the thermodynamics of life [32]. It happens that the autonomy theory is, to a large extent, a thermodynamic development of the very autopoietic theory that grounds NDT [32,34,53]. The autopoietic theory, in its classical version, lacks a thermodynamic grounding, and the autonomy theory aims to develop and update the project precisely in that regard. Considering this, the representatives of the comprehensive view might claim (a) that the autopoietic theory remains a non-teleological theory just because it remains an underdeveloped, not fully updated theoretical construction, and (b) that if this handicap was remedied, that is, if the autopoietic theory were thermodynamically updated, then it would end up by recognizing the teleological dimension of living beings, ceasing to offer a non-teleological alternative to the comprehensive NES.

In what remains, we examine this potential objection and answer it by presenting a recent, teleology-free thermodynamic elaboration of autopoiesis, namely the embodied autopoietic theory (EAT).

### 4.1. Autonomy Theory, Thermodynamics, and Teleology

Autonomy theorists observe that the cybernetic-systemic filiation of the autopoietic theory has negative implications for a genuine organism-centered biological approach. The original formulation of the autopoietic theory in the 1970s emerged in the context of cybernetics and systemic thinking, two admittedly abstract approaches to biology and cognition. Froese and Stewart, for instance, claim that “Maturana’s appropriation of the cybernetic framework in the service of an organism-centered biology is (…) misleading” ([54], p. 31). Not only that, they also claim that AT’s “blind adherence to the principles of general systems theory has made [its] conception of autopoiesis unsuitable for the foundation of an organism-centered biology” ([54], p. 39). The reason? Cybernetics and systems theory are highly abstract, formal approaches to life and cognition, and the autopoietic theory, in following them, elaborates a concept of autopoiesis that overlooks the importance of the material and thermodynamic aspects of real, concrete life. This is highly compromising because those aspects, so goes the reasoning, are precisely the key ones to identifying the distinctive nature of the living [32].

The main purpose of the autopoietic theory is to specify the distinctive nature of living beings, but this is precisely what the theory, according to this critique, fails to do.

“The problem is that if you abstract the organization of the living from its components to such an extent that you simply do not need to consider their nature at all (… whether they are thermodynamically stable or far-from-equilibrium; etc.), then you end up with a general systems concept that is no longer specifically applicable to the class of living systems”.

([54], p. 33)

We think that this observation is correct. No doubt, some degree of abstraction is useful and even perhaps necessary if a theoretical construction aims to reach some degree of generality. A theory of living beings that is too tight to the particulars of the concrete chemical realization of terrestrial life is potentially useless because too narrow; it might easily fail to cover hitherto unknown forms of life in the universe [55]. But at the same time, a too abstract theory with no reference to the minimal physical conditions under which life, whatever its form and constitution, might be possible, is equally useless; it fails to demarcate, as [54] observe, the living from the non-living. So, we agree with the autonomy theorists regarding the necessity of including and taking seriously the thermodynamic aspects of autopoiesis.

The autopoietic theory needs to be thermodynamically updated, and this is in fact what autonomy theory, according to Moreno and Mossio, claims to do:

“Maturana and Varela define autonomy in rather abstract and functionalist terms: material and energetic aspects are considered as purely contingent to its realisation. [T]he framework that we (…) develop (…) takes a different path. The autonomous perspective, we hold, should take into account (…) their “grounding” in thermodynamics”.

([32], pp. xxvii–xxviii)

However, once this is done, a new understanding of living beings appears in the view of autonomy theory. Once thermodynamics is explicitly introduced to autopoiesis, a series of emergent properties previously rejected by the autopoietic theory start to be appreciated, among which teleology comes to the fore [32,53]. Teleology, according to the autonomy theory, finds a naturalistic ground and can be therefore recognized when we attend to the thermodynamic organization of living beings as self-maintaining systems.

In the words of Moreno and Mossio, “the causal regime of a self-maintaining system provides a naturalized grounding for the teleological dimension” ([32], p. 70). By “self-maintaining systems” Moreno and Mossio mean, basically, dissipative structures such as candle flames, hurricanes and convective cells; that is, thermodynamic open systems that maintain integrity in far-from-equilibrium conditions. Living systems, being autopoietic systems, constitute a highly complex and developed version of self-maintaining systems, and therefore have, according to the theory, an intrinsic teleological dimension [33].

Returning to the objection with which this section opens, those who see in thermodynamics the ground for teleology could argue that the non-teleological character of the autopoietic theory results just from its disregard of thermodynamics. And that, conversely, if the theory were thermodynamically updated, it would end by recognizing some teleological dimension in living beings (just like the autonomy theory does), ceasing to be, through NDT, a real alternative to the comprehensive, teleologically committed version of NES.

As we said, we agree that the original formulation of the autopoietic theory was too abstract and formal, and neglected important thermodynamic aspects that must be taken into consideration. However, we do not think that the incorporation of these thermodynamic considerations affects the non-teleological character of the theory. To show this, we introduce EAT; a recent theoretical development that incorporates thermodynamics as well as keeping the non-teleological ontology of autopoiesis untouched.

### 4.2. Embodied Autopoiesis: The Thermodynamic Mark of Living Beings

Recently, representatives of the autopoietic theory, recognizing that the abstract original formulation of the theory fails to neatly demarcate the class of living beings, have offered an embodied and thermodynamic reformulation of the notion of autopoiesis that aims to distinguish living beings as a particular kind of dissipative structures [56,57]. In essence, they observe that instead of characterizing living beings as autopoietic systems, as the theory’s original formulation does, the autopoietic theory should characterize them as autopoietic bodies [56].

The proposal, labeled “embodied autopoietic theory” (EAT) [56,58], argues that the notion of system is too generic and abstract to specify the distinctive thermodynamic nature of living beings as internally cohesive, self-contained physical systems; that is, as physical bodies. It is argued that without this specification, the autopoietic theory leads us to include within the class of living beings natural systems that we should not be including. Autocatalytic networks and candle flames, for instance, are organized as recursive chemical productive networks that define a certain topology, and can therefore be qualified as autopoietic systems according to the theory’s original formulation. However, the argument continues, these systems do not keep by themselves the proximity of their products and components against the entropic tendency to spatial diffusion, as living beings distinctively do [56,59,60].

EAT states that, from the thermodynamic point of view, the distinctive feature of living beings among dissipative structures is that they resist, in a co-dependent way, two kinds of entropic tendencies at the same time: chemical decay and spatial diffusion. Agmon, who identifies autopoietic chemical network with autocatalysis, explains the idea as follows:

“The problem of decay is that molecular reactions run towards a low-energy steady state at which no spontaneous chemical activity is possible. The problem of diffusion is that due to random collisions, molecules become uniformly distributed in space and lose all system–environment separation (…). Living entities overcome these problems through the operations of autocatalysis and containment (…). Autocatalysis counters decay through a closure in catalytic reactions [and] containment (…) keeps molecules from diffusing apart. Autocatalysis and containment support each other’s function and together form an individuated, embodied autopoietic system”.

([59], p. 35)

We will not evaluate here whether this thermodynamic reformulation of autopoiesis solves or not the problem of the demarcation of living beings as a natural kind. Our purpose is not to demonstrate that EAT has the correct answer to the said problem but rather that there is a thermodynamic development of the autopoietic theory that remains consistent with the theory’s non-teleological stance.

EAT explicitly rejects “to depict living beings as agents that do things in a teleological sense” ([56], p. 57. Internal quotation marks removed). Why? For the simple reason, we take it, that there is nothing in the letter or idea of an autopoietic body that entails teleology, or that justifies its introduction within a scientific framework. On the contrary, in pointing out that among dissipative structures living beings are peculiar only in that they resist at once the entropic tendencies of chemical decay and spatial diffusion, that is, that they are at the same time chemical networks and bodies, EAT sets living beings in strict continuity with the rest of dissipative structures and natural systems in general, leaving no room to justify an exception regarding teleology.

Natural systems can be classified along a thermodynamic continuum that goes from an equilibrium condition to one that is far from it. Taking the equilibrium condition as a point of reference, we find systems that are close to it, i.e., near-equilibrium systems, and others that are far from it, i.e., far-from-equilibrium systems [61]. Whether a given system is at thermodynamic equilibrium, close to it, or far from it depends on a series of physicochemical factors such as whether the system is closed or open, and whether the system is or not exposed to some energetic gradient or field [62]. Whatever the case, the crucial point in the analysis is that all dissipative structures, including living beings, are situated within a thermodynamic continuum, with no metaphysical gap separating them from other natural systems.

Thus, as in the original ontological reasoning of the autopoietic theory (see Section 3), in EAT it can also be said that living beings, despite their peculiarity as autopoietic bodies among natural systems, remain strictly natural systems and nothing more than that. That is the case with natural systems in general, if studied according to the scientific framework, that they are not to be conceived as purposeful entities.

EAT, we conclude, holds as a thermodynamic development of the autopoietic theory that can ontologically ground a non-teleological alternative for a comprehensive NES.

## 5. Conclusions

In this paper, we showed that the autopoietic approach to evolution, i.e., NDT+EAT, is a strongly organism-centered perspective that, as such, incarnates the spirit of NES and holds as a promising option to develop the project. More specifically, we showed that NDT offers a better way to develop NES in comparison to the restrictive and comprehensive versions currently available. The restrictive version, with no explicit commitment to teleology, fits well into the scientific framework of natural science, but lacks an ontological grounding regarding the nature of organisms. The comprehensive version has such an ontological grounding, but since this is teleological, conflicts with the scientific framework. NDT, we showed, delivers the theoretical insights that are relevant for NES, and has in EAT a teleology-free ontological grounding, standing as a scientifically standard option to develop the program.

Thus, we think of the superiority of the autopoietic approach as a matter of managing to combine theoretical robustness with scientific correctness. We have not argued that the autopoietic approach is a better option because it generates novel or better predictions relative to those generated by the existing versions of NES. Assuming that it has at least a similar empirical power as the other versions of NES, our argument is that the combination NDT+EAT should be preferred because it offers a comprehensive NES without setting the project at odds with science’s methodological principles.

We defended our proposal from a potential objection regarding the theoretical change that a thermodynamic update might produce in NDT’s ontological ground. We answered the objection by appealing to EAT, which offers a thermodynamic elaboration of the autopoietic theory that, contrary to what the objection suggests, reaffirms the original ontology of the theory and holds as a non-teleological ground for a comprehensive NES.

All this, however, does not mean that we think there will be an immediate and complete integration between the autopoietic theory of evolution and NES. There are, as we saw, important and promising coincidences between these two research programs; there are also theoretical and philosophical points that pose challenges and potential conflicts for their integration. To name just one, the autopoietic theory endorses a strongly constructivist metaphysics and epistemology that takes the notion of niche construction quite beyond the reach that NES conventionally gives to it [63]. Should NES adopt or reject such a philosophical line? The answer is not obvious.

We cannot analyze this or other challenges here. We just acknowledge that much more work still needs to be done to build a theoretically consistent autopoietic version of NES.
